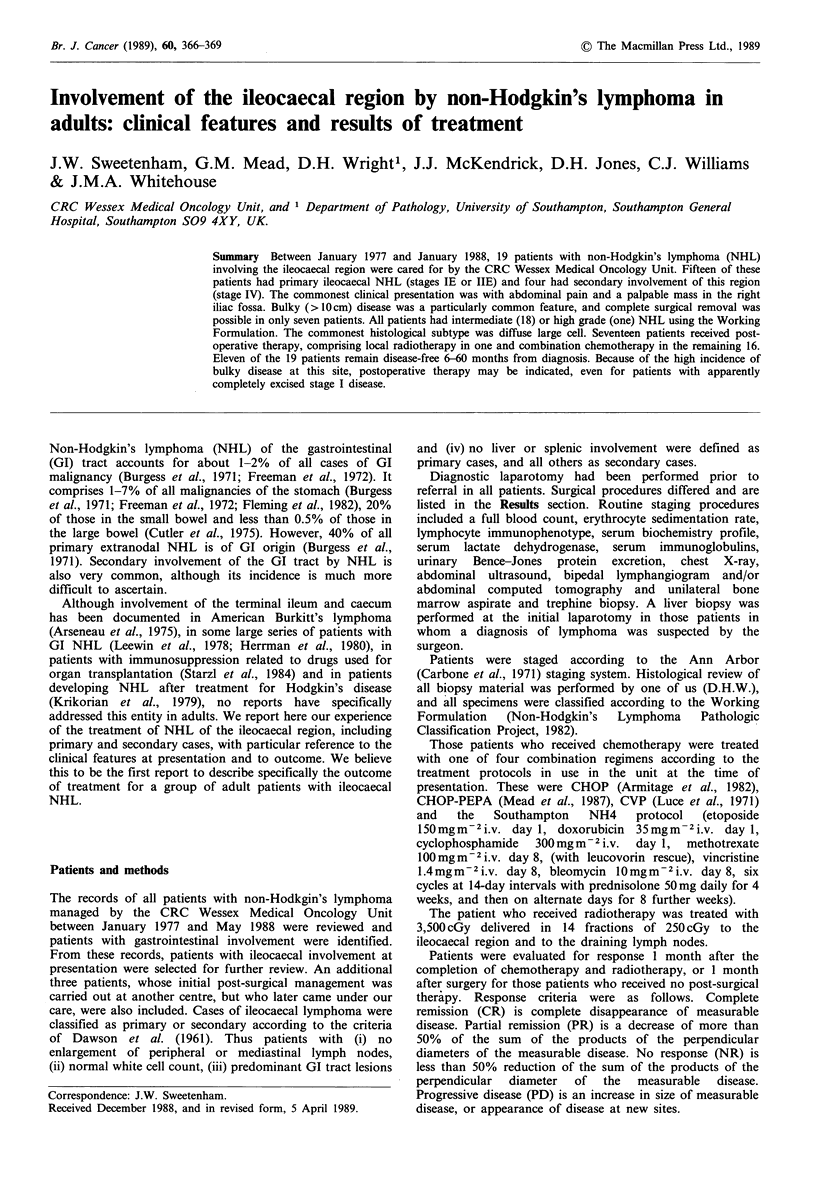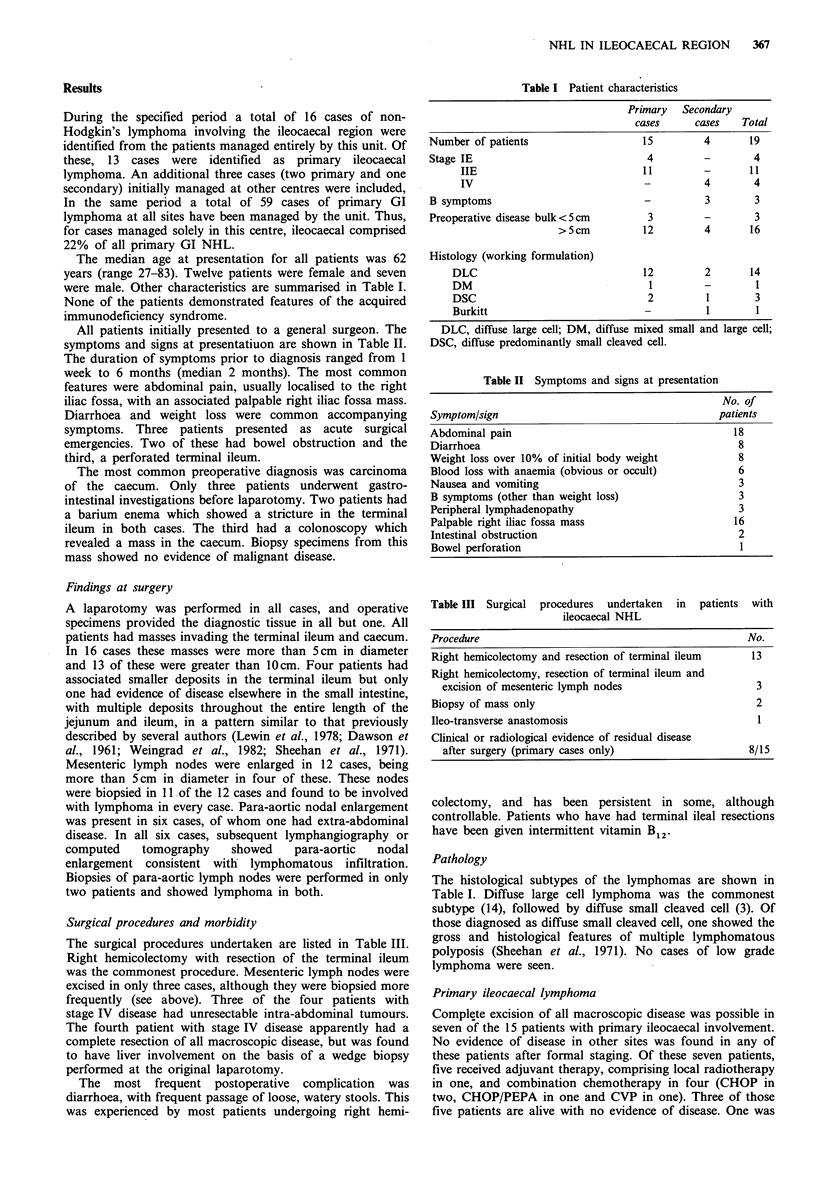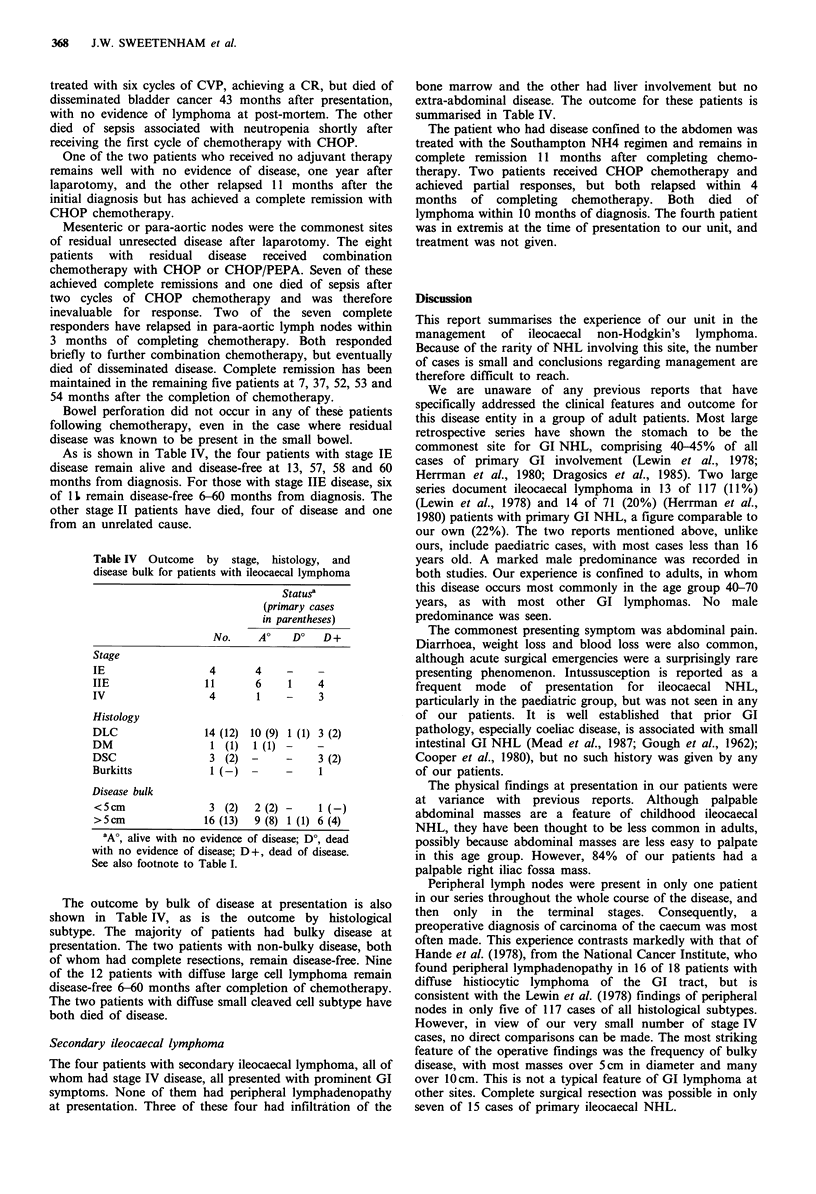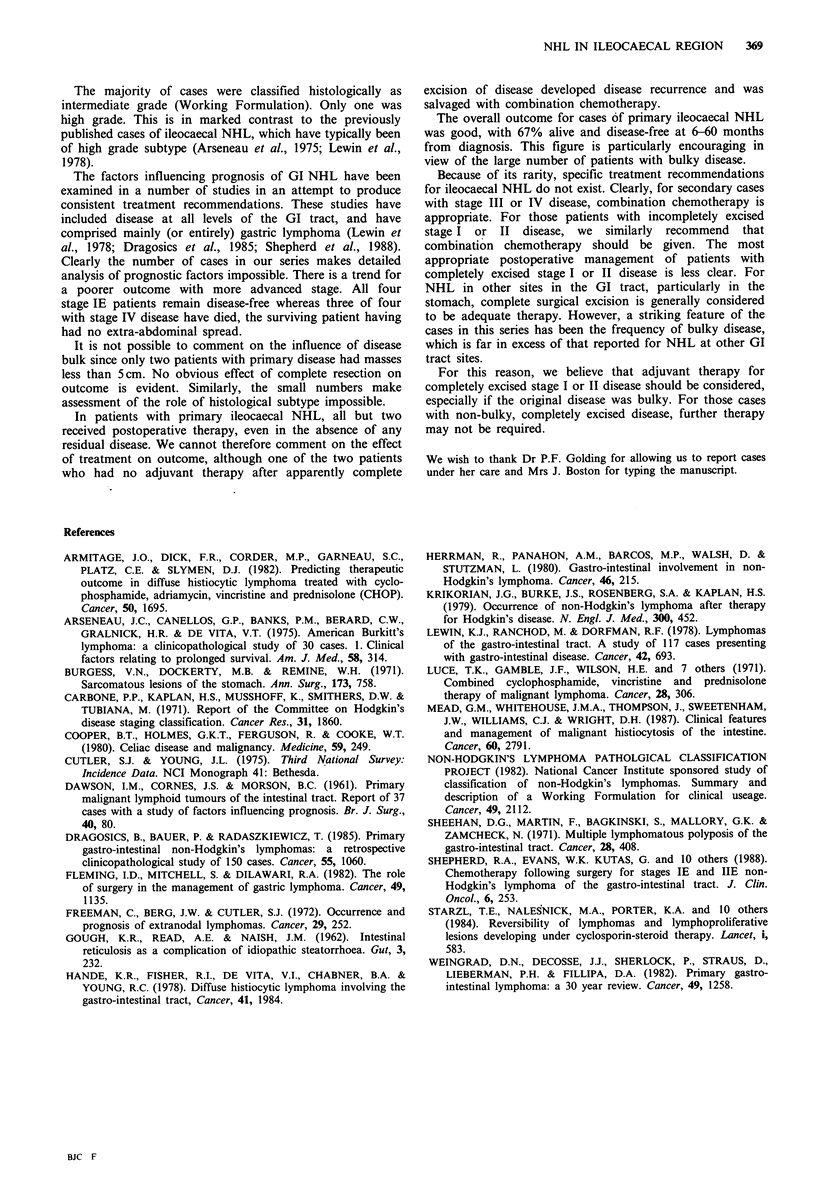# Involvement of the ileocaecal region by non-Hodgkin's lymphoma in adults: clinical features and results of treatment.

**DOI:** 10.1038/bjc.1989.286

**Published:** 1989-09

**Authors:** J. W. Sweetenham, G. M. Mead, D. H. Wright, J. J. McKendrick, D. H. Jones, C. J. Williams, J. M. Whitehouse

**Affiliations:** CRC Wessex Medical Oncology Unit, University of Southampton, UK.

## Abstract

Between January 1977 and January 1988, 19 patients with non-Hodgkin's lymphoma (NHL) involving the ileocaecal region were cared for by the CRC Wessex Medical Oncology Unit. Fifteen of these patients had primary ileocaecal NHL (stages IE or IIE) and four had secondary involvement of this region (stage IV). The commonest clinical presentation was with abdominal pain and a palpable mass in the right iliac fossa. Bulky (greater than 10 cm) disease was a particularly common feature, and complete surgical removal was possible in only seven patients. All patients had intermediate (18) or high grade (one) NHL using the Working Formulation. The commonest histological subtype was diffuse large cell. Seventeen patients received postoperative therapy, comprising local radiotherapy in one and combination chemotherapy in the remaining 16. Eleven of the 19 patients remain disease-free 6-60 months from diagnosis. Because of the high incidence of bulky disease at this site, postoperative therapy may be indicated, even for patients with apparently completely excised stage I disease.


Br. J. Cancer (1989), 60, 366–369 © The Macmillan Press Ltd., 1989

### Involvement of the ileocaecal region by non-Hodgkin's lymphoma in adults: clinical features and results of treatment

J.W. Sweetenham, G.M. Mead, D.H. Wright[1], J.J. McKendrick, D.H. Jones, C.J. Williams & J.M.A. Whitehouse

*CRC Wessex Medical Oncology Unit, and [1] Department of Pathology, University of Southampton, Southampton General Hospital, Southampton SO9 4XY, UK.*

**Summary** Between January 1977 and January 1988, 19 patients with non-Hodgkin's lymphoma (NHL) involving the ileocaecal region were cared for by the CRC Wessex Medical Oncology Unit. Fifteen of these patients had primary ileocaecal NHL (stages IE or IIE) and four had secondary involvement of this region (stage IV). The commonest clinical presentation was with abdominal pain and a palpable mass in the right iliac fossa. Bulky (>10 cm) disease was a particularly common feature, and complete surgical removal was possible in only seven patients. All patients had intermediate (18) or high grade (one) NHL using the Working Formulation. The commonest histological subtype was diffuse large cell. Seventeen patients received post-operative therapy, comprising local radiotherapy in one and combination chemotherapy in the remaining 16. Eleven of the 19 patients remain disease-free 6–60 months from diagnosis. Because of the high incidence of bulky disease at this site, postoperative therapy may be indicated, even for patients with apparently completely excised stage I disease.

Non-Hodgkin's lymphoma (NHL) of the gastrointestinal (GI) tract accounts for about 1–2% of all cases of GI malignancy (Burgess *et al.*, 1971; Freeman *et al.*, 1972). It comprises 1–7% of all malignancies of the stomach (Burgess *et al.*, 1971; Freeman *et al.*, 1972; Fleming *et al.*, 1982), 20% of those in the small bowel and less than 0.5% of those in the large bowel (Cutler *et al.*, 1975). However, 40% of all primary extranodal NHL is of GI origin (Burgess *et al.*, 1971). Secondary involvement of the GI tract by NHL is also very common, although its incidence is much more difficult to ascertain.

Although involvement of the terminal ileum and caecum has been documented in American Burkitt's lymphoma (Arseneau *et al.*, 1975), in some large series of patients with GI NHL (Leewin *et al.*, 1978; Herrman *et al.*, 1980), in patients with immunosuppression related to drugs used for organ transplantation (Starzl *et al.*, 1984) and in patients developing NHL after treatment for Hodgkin's disease (Krikorian *et al.*, 1979), no reports have specifically addressed this entity in adults. We report here our experience of the treatment of NHL of the ileocaecal region, including primary and secondary cases, with particular reference to the clinical features at presentation and to outcome. We believe this to be the first report to describe specifically the outcome of treatment for a group of adult patients with ileocaecal NHL.

### Patients and methods

The records of all patients with non-Hodkgin's lymphoma managed by the CRC Wessex Medical Oncology Unit between January 1977 and May 1988 were reviewed and patients with gastrointestinal involvement were identified. From these records, patients with ileocaecal involvement at presentation were selected for further review. An additional three patients, whose initial post-surgical management was carried out at another centre, but who later came under our care, were also included. Cases of ileocaecal lymphoma were classified as primary or secondary according to the criteria of Dawson *et al.* (1961). Thus patients with (i) no enlargement of peripheral or mediastinal lymph nodes, (ii) normal white cell count, (iii) predominant GI tract lesions and (iv) no liver or splenic involvement were defined as primary cases, and all others as secondary cases.

Diagnostic laparotomy had been performed prior to referral in all patients. Surgical procedures differed and are listed in the **Results** section. Routine staging procedures included a full blood count, erythrocyte sedimentation rate, lymphocyte immunophenotype, serum biochemistry profile, serum lactate dehydrogenase, serum immunoglobulins, urinary Bence–Jones protein excretion, chest X-ray, abdominal ultrasound, bipedal lymphangiogram and/or abdominal computed tomography and unilateral bone marrow aspirate and trephine biopsy. A liver biopsy was performed at the initial laparotomy in those patients in whom a diagnosis of lymphoma was suspected by the surgeon.

Patients were staged according to the Ann Arbor (Carbone *et al.*, 1971) staging system. Histological review of all biopsy material was performed by one of us (D.H.W.), and all specimens were classified according to the Working Formulation (Non-Hodgkin's Lymphoma Pathologic Classification Project, 1982).

Those patients who received chemotherapy were treated with one of four combination regimens according to the treatment protocols in use in the unit at the time of presentation. These were CHOP (Armitage *et al.*, 1982), CHOP-PEPA (Mead *et al.*, 1987), CVP (Luce *et al.*, 1971) and the Southampton NH4 protocol (etoposide $150\,mg\,m^{-2}$ i.v. day 1, doxorubicin $35\,mg\,m^{-2}$ i.v. day 1, cyclophosphamide $300\,mg\,m^{-2}$ i.v. day 1, methotrexate $100\,mg\,m^{-2}$ i.v. day 8, (with leucovorin rescue), vincristine $1.4\,mg\,m^{-2}$ i.v. day 8, bleomycin $10\,mg\,m^{-2}$ i.v. day 8, six cycles at 14-day intervals with prednisolone 50 mg daily for 4 weeks, and then on alternate days for 8 further weeks).

The patient who received radiotherapy was treated with 3,500 cGy delivered in 14 fractions of 250 cGy to the ileocaecal region and to the draining lymph nodes.

Patients were evaluated for response 1 month after the completion of chemotherapy and radiotherapy, or 1 month after surgery for those patients who received no post-surgical therapy. Response criteria were as follows. Complete remission (CR) is complete disappearance of measurable disease. Partial remission (PR) is a decrease of more than 50% of the sum of the products of the perpendicular diameters of the measurable disease. No response (NR) is less than 50% reduction of the sum of the products of the perpendicular diameter of the measurable disease. Progressive disease (PD) is an increase in size of measurable disease, or appearance of disease at new sites.

Correspondence: J.W. Sweetenham.
Received December 1988, and in revised form, 5 April 1989.

### Results

During the specified period a total of 16 cases of non-Hodgkin's lymphoma involving the ileocaecal region were identified from the patients managed entirely by this unit. Of these, 13 cases were identified as primary ileocaecal lymphoma. An additional three cases (two primary and one secondary) initially managed at other centres were included, In the same period a total of 59 cases of primary GI lymphoma at all sites have been managed by the unit. Thus, for cases managed solely in this centre, ileocaecal comprised 22% of all primary GI NHL.

The median age at presentation for all patients was 62 years (range 27–83). Twelve patients were female and seven were male. Other characteristics are summarised in Table I. None of the patients demonstrated features of the acquired immunodeficiency syndrome.

All patients initially presented to a general surgeon. The symptoms and signs at presentatiuon are shown in Table II. The duration of symptoms prior to diagnosis ranged from 1 week to 6 months (median 2 months). The most common features were abdominal pain, usually localised to the right iliac fossa, with an associated palpable right iliac fossa mass. Diarrhoea and weight loss were common accompanying symptoms. Three patients presented as acute surgical emergencies. Two of these had bowel obstruction and the third, a perforated terminal ileum.

The most common preoperative diagnosis was carcinoma of the caecum. Only three patients underwent gastrointestinal investigations before laparotomy. Two patients had a barium enema which showed a stricture in the terminal ileum in both cases. The third had a colonoscopy which revealed a mass in the caecum. Biopsy specimens from this mass showed no evidence of malignant disease.

**Table I** Patient characteristics

| | *Primary cases* | *Secondary cases* | *Total* |
|---|---|---|---|
| Number of patients | 15 | 4 | 19 |
| Stage IE | 4 | – | 4 |
| IIE | 11 | – | 11 |
| IV | – | 4 | 4 |
| B symptoms | – | 3 | 3 |
| Preoperative disease bulk < 5 cm | 3 | – | 3 |
| > 5 cm | 12 | 4 | 16 |
| Histology (working formulation) | | | |
| DLC | 12 | 2 | 14 |
| DM | 1 | – | 1 |
| DSC | 2 | 1 | 3 |
| Burkitt | – | 1 | 1 |

DLC, diffuse large cell; DM, diffuse mixed small and large cell; DSC, diffuse predominantly small cleaved cell.

**Table II** Symptoms and signs at presentation

| *Symptom/sign* | *No. of patients* |
|---|---|
| Abdominal pain | 18 |
| Diarrhoea | 8 |
| Weight loss over 10% of initial body weight | 8 |
| Blood loss with anaemia (obvious or occult) | 6 |
| Nausea and vomiting | 3 |
| B symptoms (other than weight loss) | 3 |
| Peripheral lymphadenopathy | 3 |
| Palpable right iliac fossa mass | 16 |
| Intestinal obstruction | 2 |
| Bowel perforation | 1 |

### *Findings at surgery*

A laparotomy was performed in all cases, and operative specimens provided the diagnostic tissue in all but one. All patients had masses invading the terminal ileum and caecum. In 16 cases these masses were more than 5 cm in diameter and 13 of these were greater than 10 cm. Four patients had associated smaller deposits in the terminal ileum but only one had evidence of disease elsewhere in the small intestine, with multiple deposits throughout the entire length of the jejunum and ileum, in a pattern similar to that previously described by several authors (Lewin *et al.*, 1978; Dawson *et al.*, 1961; Weingrad *et al.*, 1982; Sheehan *et al.*, 1971). Mesenteric lymph nodes were enlarged in 12 cases, being more than 5 cm in diameter in four of these. These nodes were biopsied in 11 of the 12 cases and found to be involved with lymphoma in every case. Para-aortic nodal enlargement was present in six cases, of whom one had extra-abdominal disease. In all six cases, subsequent lymphangiography or computed tomography showed para-aortic nodal enlargement consistent with lymphomatous infiltration. Biopsies of para-aortic lymph nodes were performed in only two patients and showed lymphoma in both.

### *Surgical procedures and morbidity*

The surgical procedures undertaken are listed in Table III. Right hemicolectomy with resection of the terminal ileum was the commonest procedure. Mesenteric lymph nodes were excised in only three cases, although they were biopsied more frequently (see above). Three of the four patients with stage IV disease had unresectable intra-abdominal tumours. The fourth patient with stage IV disease apparently had a complete resection of all macroscopic disease, but was found to have liver involvement on the basis of a wedge biopsy performed at the original laparotomy.

The most frequent postoperative complication was diarrhoea, with frequent passage of loose, watery stools. This was experienced by most patients undergoing right hemicolectomy, and has been persistent in some, although controllable. Patients who have had terminal ileal resections have been given intermittent vitamin $B_{12}$.

**Table III** Surgical procedures undertaken in patients with ileocaecal NHL

| *Procedure* | *No.* |
|---|---|
| Right hemicolectomy and resection of terminal ileum | 13 |
| Right hemicolectomy, resection of terminal ileum and excision of mesenteric lymph nodes | 3 |
| Biopsy of mass only | 2 |
| Ileo-transverse anastomosis | 1 |
| Clinical or radiological evidence of residual disease after surgery (primary cases only) | 8/15 |

### *Pathology*

The histological subtypes of the lymphomas are shown in Table I. Diffuse large cell lymphoma was the commonest subtype (14), followed by diffuse small cleaved cell (3). Of those diagnosed as diffuse small cleaved cell, one showed the gross and histological features of multiple lymphomatous polyposis (Sheehan *et al.*, 1971). No cases of low grade lymphoma were seen.

### *Primary ileocaecal lymphoma*

Complete excision of all macroscopic disease was possible in seven of the 15 patients with primary ileocaecal involvement. No evidence of disease in other sites was found in any of these patients after formal staging. Of these seven patients, five received adjuvant therapy, comprising local radiotherapy in one, and combination chemotherapy in four (CHOP in two, CHOP/PEPA in one and CVP in one). Three of those five patients are alive with no evidence of disease. One was treated with six cycles of CVP, achieving a CR, but died of disseminated bladder cancer 43 months after presentation, with no evidence of lymphoma at post-mortem. The other died of sepsis associated with neutropenia shortly after receiving the first cycle of chemotherapy with CHOP.

One of the two patients who received no adjuvant therapy remains well with no evidence of disease, one year after laparotomy, and the other relapsed 11 months after the initial diagnosis but has achieved a complete remission with CHOP chemotherapy.

Mesenteric or para-aortic nodes were the commonest sites of residual unresected disease after laparotomy. The eight patients with residual disease received combination chemotherapy with CHOP or CHOP/PEPA. Seven of these achieved complete remissions and one died of sepsis after two cycles of CHOP chemotherapy and was therefore inevaluable for response. Two of the seven complete responders have relapsed in para-aortic lymph nodes within 3 months of completing chemotherapy. Both responded briefly to further combination chemotherapy, but eventually died of disseminated disease. Complete remission has been maintained in the remaining five patients at 7, 37, 52, 53 and 54 months after the completion of chemotherapy.

Bowel perforation did not occur in any of these patients following chemotherapy, even in the case where residual disease was known to be present in the small bowel.

As is shown in Table IV, the four patients with stage IE disease remain alive and disease-free at 13, 57, 58 and 60 months from diagnosis. For those with stage IIE disease, six of 11 remain disease-free 6–60 months from diagnosis. The other stage II patients have died, four of disease and one from an unrelated cause.

**Table IV** Outcome by stage, histology, and disease bulk for patients with ileocaecal lymphoma

| | | *Status*[a] *(primary cases in parentheses)* | | |
|---|---|---|---|---|
| | *No.* | *A°* | *D°* | *D+* |
| *Stage* | | | | |
| IE | 4 | 4 | – | – |
| IIE | 11 | 6 | 1 | 4 |
| IV | 4 | 1 | – | 3 |
| *Histology* | | | | |
| DLC | 14 (12) | 10 (9) | 1 (1) | 3 (2) |
| DM | 1 (1) | 1 (1) | – | – |
| DSC | 3 (2) | – | – | 3 (2) |
| Burkitts | 1 (–) | – | – | 1 |
| *Disease bulk* | | | | |
| <5 cm | 3 (2) | 2 (2) | – | 1 (–) |
| >5 cm | 16 (13) | 9 (8) | 1 (1) | 6 (4) |

[a]A°, alive with no evidence of disease; D°, dead with no evidence of disease; D+, dead of disease. See also footnote to Table I.

The outcome by bulk of disease at presentation is also shown in Table IV, as is the outcome by histological subtype. The majority of patients had bulky disease at presentation. The two patients with non-bulky disease, both of whom had complete resections, remain disease-free. Nine of the 12 patients with diffuse large cell lymphoma remain disease-free 6–60 months after completion of chemotherapy. The two patients with diffuse small cleaved cell subtype have both died of disease.

*Secondary ileocaecal lymphoma*

The four patients with secondary ileocaecal lymphoma, all of whom had stage IV disease, all presented with prominent GI symptoms. None of them had peripheral lymphadenopathy at presentation. Three of these four had infiltration of the bone marrow and the other had liver involvement but no extra-abdominal disease. The outcome for these patients is summarised in Table IV.

The patient who had disease confined to the abdomen was treated with the Southampton NH4 regimen and remains in complete remission 11 months after completing chemotherapy. Two patients received CHOP chemotherapy and achieved partial responses, but both relapsed within 4 months of completing chemotherapy. Both died of lymphoma within 10 months of diagnosis. The fourth patient was in extremis at the time of presentation to our unit, and treatment was not given.

### Discussion

This report summarises the experience of our unit in the management of ileocaecal non-Hodgkin's lymphoma. Because of the rarity of NHL involving this site, the number of cases is small and conclusions regarding management are therefore difficult to reach.

We are unaware of any previous reports that have specifically addressed the clinical features and outcome for this disease entity in a group of adult patients. Most large retrospective series have shown the stomach to be the commonest site for GI NHL, comprising 40–45% of all cases of primary GI involvement (Lewin *et al.*, 1978; Herrman *et al.*, 1980; Dragosics *et al.*, 1985). Two large series document ileocaecal lymphoma in 13 of 117 (11%) (Lewin *et al.*, 1978) and 14 of 71 (20%) (Herrman *et al.*, 1980) patients with primary GI NHL, a figure comparable to our own (22%). The two reports mentioned above, unlike ours, include paediatric cases, with most cases less than 16 years old. A marked male predominance was recorded in both studies. Our experience is confined to adults, in whom this disease occurs most commonly in the age group 40–70 years, as with most other GI lymphomas. No male predominance was seen.

The commonest presenting symptom was abdominal pain. Diarrhoea, weight loss and blood loss were also common, although acute surgical emergencies were a surprisingly rare presenting phenomenon. Intussusception is reported as a frequent mode of presentation for ileocaecal NHL, particularly in the paediatric group, but was not seen in any of our patients. It is well established that prior GI pathology, especially coeliac disease, is associated with small intestinal GI NHL (Mead *et al.*, 1987; Gough *et al.*, 1962); Cooper *et al.*, 1980), but no such history was given by any of our patients.

The physical findings at presentation in our patients were at variance with previous reports. Although palpable abdominal masses are a feature of childhood ileocaecal NHL, they have been thought to be less common in adults, possibly because abdominal masses are less easy to palpate in this age group. However, 84% of our patients had a palpable right iliac fossa mass.

Peripheral lymph nodes were present in only one patient in our series throughout the whole course of the disease, and then only in the terminal stages. Consequently, a preoperative diagnosis of carcinoma of the caecum was most often made. This experience contrasts markedly with that of Hande *et al.* (1978), from the National Cancer Institute, who found peripheral lymphadenopathy in 16 of 18 patients with diffuse histiocytic lymphoma of the GI tract, but is consistent with the Lewin *et al.* (1978) findings of peripheral nodes in only five of 117 cases of all histological subtypes. However, in view of our very small number of stage IV cases, no direct comparisons can be made. The most striking feature of the operative findings was the frequency of bulky disease, with most masses over 5 cm in diameter and many over 10 cm. This is not a typical feature of GI lymphoma at other sites. Complete surgical resection was possible in only seven of 15 cases of primary ileocaecal NHL.

The majority of cases were classified histologically as intermediate grade (Working Formulation). Only one was high grade. This is in marked contrast to the previously published cases of ileocaecal NHL, which have typically been of high grade subtype (Arseneau *et al.*, 1975; Lewin *et al.*, 1978).

The factors influencing prognosis of GI NHL have been examined in a number of studies in an attempt to produce consistent treatment recommendations. These studies have included disease at all levels of the GI tract, and have comprised mainly (or entirely) gastric lymphoma (Lewin *et al.*, 1978; Dragosics *et al.*, 1985; Shepherd *et al.*, 1988). Clearly the number of cases in our series makes detailed analysis of prognostic factors impossible. There is a trend for a poorer outcome with more advanced stage. All four stage IE patients remain disease-free whereas three of four with stage IV disease have died, the surviving patient having had no extra-abdominal spread.

It is not possible to comment on the influence of disease bulk since only two patients with primary disease had masses less than 5 cm. No obvious effect of complete resection on outcome is evident. Similarly, the small numbers make assessment of the role of histological subtype impossible.

In patients with primary ileocaecal NHL, all but two received postoperative therapy, even in the absence of any residual disease. We cannot therefore comment on the effect of treatment on outcome, although one of the two patients who had no adjuvant therapy after apparently complete excision of disease developed disease recurrence and was salvaged with combination chemotherapy.

The overall outcome for cases of primary ileocaecal NHL was good, with 67% alive and disease-free at 6–60 months from diagnosis. This figure is particularly encouraging in view of the large number of patients with bulky disease.

Because of its rarity, specific treatment recommendations for ileocaecal NHL do not exist. Clearly, for secondary cases with stage III or IV disease, combination chemotherapy is appropriate. For those patients with incompletely excised stage I or II disease, we similarly recommend that combination chemotherapy should be given. The most appropriate postoperative management of patients with completely excised stage I or II disease is less clear. For NHL in other sites in the GI tract, particularly in the stomach, complete surgical excision is generally considered to be adequate therapy. However, a striking feature of the cases in this series has been the frequency of bulky disease, which is far in excess of that reported for NHL at other GI tract sites.

For this reason, we believe that adjuvant therapy for completely excised stage I or II disease should be considered, especially if the original disease was bulky. For those cases with non-bulky, completely excised disease, further therapy may not be required.

We wish to thank Dr P.F. Golding for allowing us to report cases under her care and Mrs J. Boston for typing the manuscript.

### References

ARMITAGE, J.O., DICK, F.R., CORDER, M.P., GARNEAU, S.C., PLATZ, C.E. & SLYMEN, D.J. (1982). Predicting therapeutic outcome in diffuse histiocytic lymphoma treated with cyclophosphamide, adriamycin, vincristine and prednisolone (CHOP). *Cancer*, **50**, 1695.

ARSENEAU, J.C., CANELLOS, G.P., BANKS, P.M., BERARD, C.W., GRALNICK, H.R. & DE VITA, V.T. (1975). American Burkitt's lymphoma: a clinicopathological study of 30 cases. 1. Clinical factors relating to prolonged survival. *Am. J. Med.*, **58**, 314.

BURGESS, V.N., DOCKERTY, M.B. & REMINE, W.H. (1971). Sarcomatous lesions of the stomach. *Ann. Surg.*, **173**, 758.

CARBONE, P.P., KAPLAN, H.S., MUSSHOFF, K., SMITHERS, D.W. & TUBIANA, M. (1971). Report of the Committee on Hodgkin's disease staging classification. *Cancer Res.*, **31**, 1860.

COOPER, B.T., HOLMES, G.K.T., FERGUSON, R. & COOKE, W.T. (1980). Celiac disease and malignancy. *Medicine*, **59**, 249.

CUTLER, S.J. & YOUNG, J.L. (1975). *Third National Survey: Incidence Data*. NCI Monograph 41: Bethesda.

DAWSON, I.M., CORNES, J.S. & MORSON, B.C. (1961). Primary malignant lymphoid tumours of the intestinal tract. Report of 37 cases with a study of factors influencing prognosis. *Br. J. Surg.*, **40**, 80.

DRAGOSICS, B., BAUER, P. & RADASZKIEWICZ, T. (1985). Primary gastro-intestinal non-Hodgkin's lymphomas: a retrospective clinicopathological study of 150 cases. *Cancer*, **55**, 1060.

FLEMING, I.D., MITCHELL, S. & DILAWARI, R.A. (1982). The role of surgery in the management of gastric lymphoma. *Cancer*, **49**, 1135.

FREEMAN, C., BERG, J.W. & CUTLER, S.J. (1972). Occurrence and prognosis of extranodal lymphomas. *Cancer*, **29**, 252.

GOUGH, K.R., READ, A.E. & NAISH, J.M. (1962). Intestinal reticulosis as a complication of idiopathic steatorrhoea. *Gut*, **3**, 232.

HANDE, K.R., FISHER, R.I., DE VITA, V.I., CHABNER, B.A. & YOUNG, R.C. (1978). Diffuse histiocytic lymphoma involving the gastro-intestinal tract, *Cancer*, **41**, 1984.

HERRMAN, R., PANAHON, A.M., BARCOS, M.P., WALSH, D. & STUTZMAN, L. (1980). Gastro-intestinal involvement in non-Hodgkin's lymphoma. *Cancer*, **46**, 215.

KRIKORIAN, J.G., BURKE, J.S., ROSENBERG, S.A. & KAPLAN, H.S. (1979). Occurrence of non-Hodgkin's lymphoma after therapy for Hodgkin's disease. *N. Engl. J. Med.*, **300**, 452.

LEWIN, K.J., RANCHOD, M. & DORFMAN, R.F. (1978). Lymphomas of the gastro-intestinal tract. A study of 117 cases presenting with gastro-intestinal disease. *Cancer*, **42**, 693.

LUCE, T.K., GAMBLE, J.F., WILSON, H.E. and 7 others (1971). Combined cyclophosphamide, vincristine and prednisolone therapy of malignant lymphoma. *Cancer*, **28**, 306.

MEAD, G.M., WHITEHOUSE, J.M.A., THOMPSON, J., SWEETENHAM, J.W., WILLIAMS, C.J. & WRIGHT, D.H. (1987). Clinical features and management of malignant histiocytosis of the intestine. *Cancer*, **60**, 2791.

NON-HODGKIN'S LYMPHOMA PATHOLGICAL CLASSIFICATION PROJECT (1982). National Cancer Institute sponsored study of classification of non-Hodgkin's lymphomas. Summary and description of a Working Formulation for clinical useage. *Cancer*, **49**, 2112.

SHEEHAN, D.G., MARTIN, F., BAGKINSKI, S., MALLORY, G.K. & ZAMCHECK, N. (1971). Multiple lymphomatous polyposis of the gastro-intestinal tract. *Cancer*, **28**, 408.

SHEPHERD, R.A., EVANS, W.K. KUTAS, G. and 10 others (1988). Chemotherapy following surgery for stages IE and IIE non-Hodgkin's lymphoma of the gastro-intestinal tract. *J. Clin. Oncol.*, **6**, 253.

STARZL, T.E., NALEŚNICK, M.A., PORTER, K.A. and 10 others (1984). Reversibility of lymphomas and lymphoproliferative lesions developing under cyclosporin-steroid therapy. *Lancet*, **i**, 583.

WEINGRAD, D.N., DECOSSE, J.J., SHERLOCK, P., STRAUS, D., LIEBERMAN, P.H. & FILLIPA, D.A. (1982). Primary gastro-intestinal lymphoma: a 30 year review. *Cancer*, **49**, 1258.